# Propofol (2,6‐diisopropylphenol) is an applicable immersion anesthetic in the axolotl with potential uses in hemodynamic and neurophysiological experiments

**DOI:** 10.1002/reg2.80

**Published:** 2017-07-27

**Authors:** Mathias Møller Thygesen, Mikkel Mylius Rasmussen, Jesper Guldsmed Madsen, Michael Pedersen, Henrik Lauridsen

**Affiliations:** ^1^ Comparative Medicine Laboratory Department of Clinical Medicine, Aarhus University Palle Juul‐Jensens Boulevard 99 8200 Aarhus N Denmark; ^2^ Department of Neurosurgery Aarhus University Hospital Noerrebrogade 44 8000 Aarhus C Denmark; ^3^ Cardiovascular Developmental Bioengineering Laboratory, Nancy E. and Peter C. Meinig School of Biomedical Engineering Cornell University 304 Weill Hall Ithaca NY 14853‐7202 USA

**Keywords:** *Ambystoma mexicanum*, anesthesia, evoked motor potentials, hemodynamic, propofol, regeneration

## Abstract

The Mexican axolotl (*Ambystoma mexicanum*) is an important model species in regenerative biology. Traditionally, axolotls are anesthetized using benzocaine or MS‐222, both of which act to inhibit voltage gated sodium channels thereby preventing action potential propagation. In some neurophysiological experiments this is not desirable; therefore we tested propofol as an alternative anesthetic in the axolotl. We evaluated benzocaine, MS‐222, and propofol's cardiovascular effects, effects on action potential propagation in the spinal cord, and gross limb regenerative effects. We found that propofol is applicable as a general anesthetic in the axolotl allowing for neurophysiological experiments and yielding a stable anesthesia with significantly less cardiovascular effect than both benzocaine and MS‐222. Additionally, propofol did not affect gross limb regeneration. In conclusion we suggest the consideration of propofol as an alternative immersion anesthetic to benzocaine and MS‐222.

## 1 INTRODUCTION

The Mexican axolotl (*Ambystoma mexicanum*) has an impressive regenerative potential and has been applied as a model for intrinsic tissue regeneration of various organs and tissues (Armstrong & Malacinski, 1989; McCusker & Gardiner, 2011; Roy & Gatien, 2008). Traditionally, intervention studies in this species have been conducted using benzocaine (ethyl 4‐aminobenzoate; other generic names Anästhesin, Americaine) or MS‐222 (ethyl 3‐aminobenzoate methanesulfonic acid; other generic names tricaine; tricaine mesylate, tricaine methanesulfonate, TMS, metacaine) as the preferred anesthetic (Diaz Quiroz, Tsai, Coyle, Sehm, & Echeverri, 2014; Khattak et al., 2014; McCusker & Gardiner, 2013; McHedlishvili, Mazurov, & Tanaka, 2012). General anesthesia is obtained by immersing the axolotl in a buffered aqueous solution of the selected anesthetic that is absorbed across the gills and the highly permeable skin. The depth and duration of anesthesia are controlled by regulating the concentration of the active compound and the exposure time. In our experience, axolotls can be kept under anesthesia for several hours allowing for complex surgical and imaging procedures with no subsequent adverse effects related to the anesthetic.

Both benzocaine and MS‐222 act to inhibit the axonal voltage gated sodium channels, thereby preventing or reducing the propagation of action potentials in the nervous system (Baker, 2000; Butler & Ward, 1965; Chevallier, Landry, Nagy, & Cabelguen, 2004; Guven, Mert, & Gunay, 2005). It follows that these types of anesthetics have obvious drawbacks when performing electrophysiological experiments on sedated animals, since the conduction pathways are inhibited during anesthesia. Inspired by the need to measure the presence/absence of conduction in the regenerating spinal cord of the axolotl under anesthesia, we set out to find an alternative to the traditional anesthesia regimes used for this animal and to test the applicability of this alternative in relevant situations for the use of the animal.

Propofol (2,6‐diisopropylphenol) is an intravenous short‐acting anesthetic widely used for animal and human surgery and other sedative purposes. Importantly, propofol is used in clinical neurosurgery combined with measurements of evoked motor potential (EMP) (Malcharek et al., 2014), making it a prime candidate for similar procedures in the axolotl. In humans, propofol exerts its effect through potentiation of the GABAa receptor activity (Kawaguchi, Furuya, & Patel, 2005), enhancing the sleep effect of GABA release in the posterior hypothalamus (Nitz & Siegel, 1996). Interestingly, propofol does not invoke a universal response throughout the central nervous system (CNS) but has localized effects on hypothalamic pathways.

To our knowledge, propofol has not previously been applied as an anesthetic on the axolotl, and its use in amphibians is limited. One study, however, has documented successful induction and management of anesthesia in the aquatic African clawed frog (*Xenopus laevis*) using 88 mg/L propofol in aquarium water, indicating that propofol diffuses over the semipermeable amphibian skin (Guenette, Beaudry, & Vachon, 2008).

In this study, we investigated the applicability of propofol as an immersion anesthetic in the axolotl and its feasibility in electrophysiological experiments without diminishing the axonal conduction in the spinal cord. Keeping in mind that the axolotl is a prime model of limb regeneration (McCusker & Gardiner, 2011; Roy & Gatien, 2008) and is emerging as a model of heart regeneration (Cano‐Martinez et al., 2010; Nakamura et al., 2016), we also tested how repeated exposures to propofol anesthesia affect gross anatomical limb regeneration and how propofol anesthesia affects cardiac function.

## 2 RESULTS

### 2.1 Effect of benzocaine, MS‐222, and propofol on cardiovascular function

The baseline heart rates (HRs) did not differ significantly between the anesthesia regimes: 24.39 ± 3.42 beats per minute (BPM), 27.33 ± 2.28 BPM, and 26.47 ± 7.81 BPM for benzocaine, MS‐222, and propofol, respectively. Likewise, baseline stroke volumes (SVs) were not significantly different between anesthesia regimes: 24.56 ± 11.78 μL, 30.29 ± 8.53 μL, and 28.34 ± 6.31 μL for benzocaine, MS‐222, and propofol, respectively.

**Figure 1:**
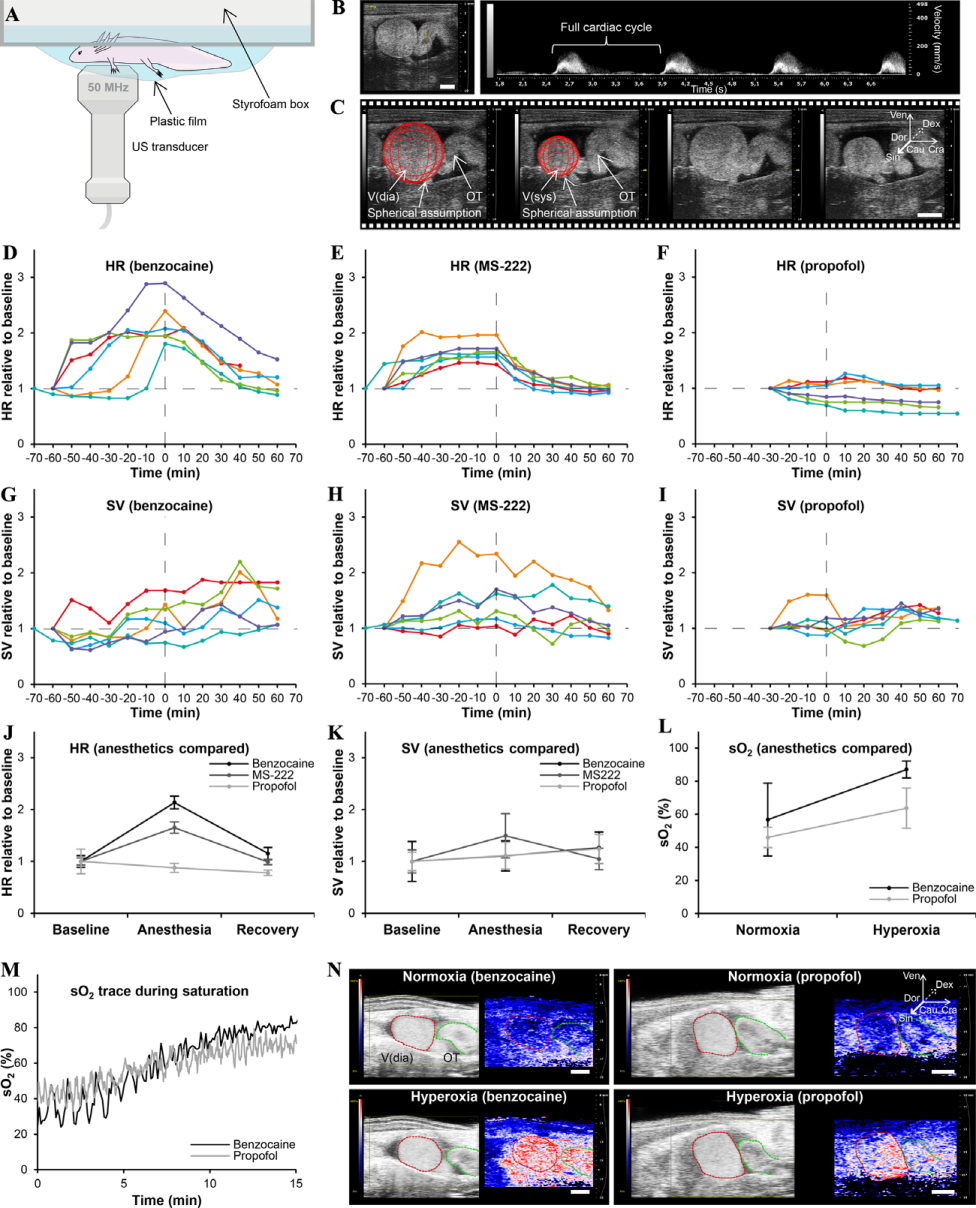
**Effect of benzocaine, MS‐222, and propofol on cardiovascular function. (**A) Experimental TTE setup. (B) Pulsed wave velocity TTE allows for HR measurements (panel to the left shows image probe position). (C) Brightness mode TTE allows for SV measurements assuming a spherical shape of the ventricle. (D)−(F) HR plotted over time (0 h is at full anesthesia) for all animals for benzocaine (D), MS‐222 (E), and propofol (F). (G)−(I) SV plotted over time for all animals for benzocaine (G), MS‐222 (H), and propofol (H). (J)−(K) Comparison of mean HR (J) and SV (K) for the three anesthetics at baseline, full anesthesia, and full recovery. (L) Mean intracardiac blood oxygen saturation (sO_2_) at normoxia and 100% ambient oxygen saturation for benzocaine and propofol. (M), (N) Representative sO_2_ traces (M) and sO_2_ images (N) during ambient oxygen saturation for benzocaine and propofol. V(dia), ventricle in diastole; V(sys), ventricle in systole; Ven, ventral; Dor, dorsal; Cau, caudal; Cra, cranial; Dex, right; Sin, left

Both benzocaine and MS‐222 yielded complete anesthesia of all axolotls after 60 min of exposure, except for one animal which met the criteria after 70 min in both cases. All animals recovered fully after 60 min in anesthetic‐free water. The HRs at full anesthesia significantly increased 2.13‐fold (95% confidence interval [CI] 0.12, *p* < 0.01) for benzocaine and 1.65‐fold (95% CI 0.11, *p* < 0.01) for MS‐222, compared to baseline HRs (Fig. 1D, E and J). HRs at baseline and after recovery were not significantly different (Fig. 1D, E and J). Benzocaine had no significant effect on SV during anesthesia, but it significantly increased SV by 1.26‐fold (95% CI 0.30, *p* < 0.5) after recovery compared to baseline (Fig. 1G, K). In contrast, MS‐222 resulted in a significant 1.49‐fold (95% CI 0.43, *p* < 0.5) increase in SV during anesthesia, but SV returned to baseline level after recovery (Fig. 1H, K).

Propofol provided complete anesthesia after 30 min in all animals, and complete recovery was recognized after 60 min except for one animal, which met the criteria after 70 min. HR was unchanged during anesthesia and after recovery (Fig. 1F, J). Propofol had no effect on SV during anesthesia, but SV was significantly elevated by 1.24‐fold (95% CI 0.28, *p* < 0.01) after recovery (Fig. 1I, K).

Blood oxygen saturation in the ventricle at normoxic conditions was not significantly different between benzocaine and propofol anesthetized animals (Fig. 1L). However, following 15 min exposure of hyperoxia (100% O_2_ saturated ambient water), the intracardiac oxygen saturation in benzocaine anesthetized animals was significantly higher than in propofol anesthetized animals (Fig. 1L, M and N).

### 2.2 Effect of benzocaine, MS‐222, and propofol on evoked motor potentials

**Figure 2:**
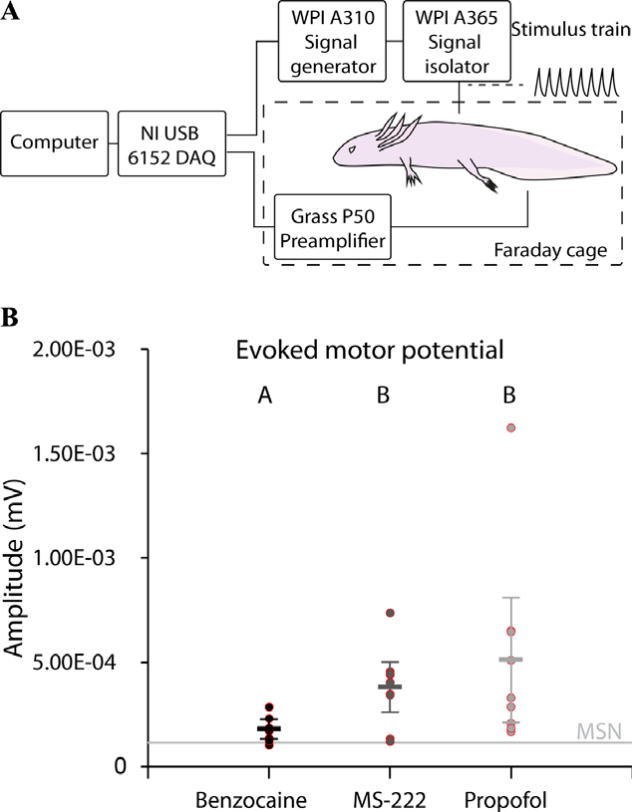
**Effect of benzocaine, MS‐222, and propofol on evoked motor potentials**. (A) Schematic illustration of the experimental EMP setup. (B) Peak‐to‐peak amplitudes of EMP using different anesthetics. EMP amplitude was significantly different between propofol and benzocaine, and between MS‐222 and benzocaine (indicated by different lettering, A and B, over data points). There was no significant difference between propofol and MS‐222. MSN, mean signal noise, showing the mean background noise level

EMP response amplitudes for benzocaine anesthetized animals were markedly lower than those recorded for propofol and MS‐222 (Fig. 2). EMP amplitude was found to be significantly different between propofol and benzocaine, and between MS‐222 and benzocaine. However, there was no significant difference between propofol and MS‐222. There seems to be a higher degree of variability in EMP amplitude using propofol.

Compared with animals anesthetized with benzocaine or propofol several animals anesthetized with MS‐222 showed signs of less deep anesthesia during the experiments, manifested as involuntary movements during measurements.

### 2.3 Effect of benzocaine, MS‐222, and propofol on gross limb regeneration

**Figure 3:**
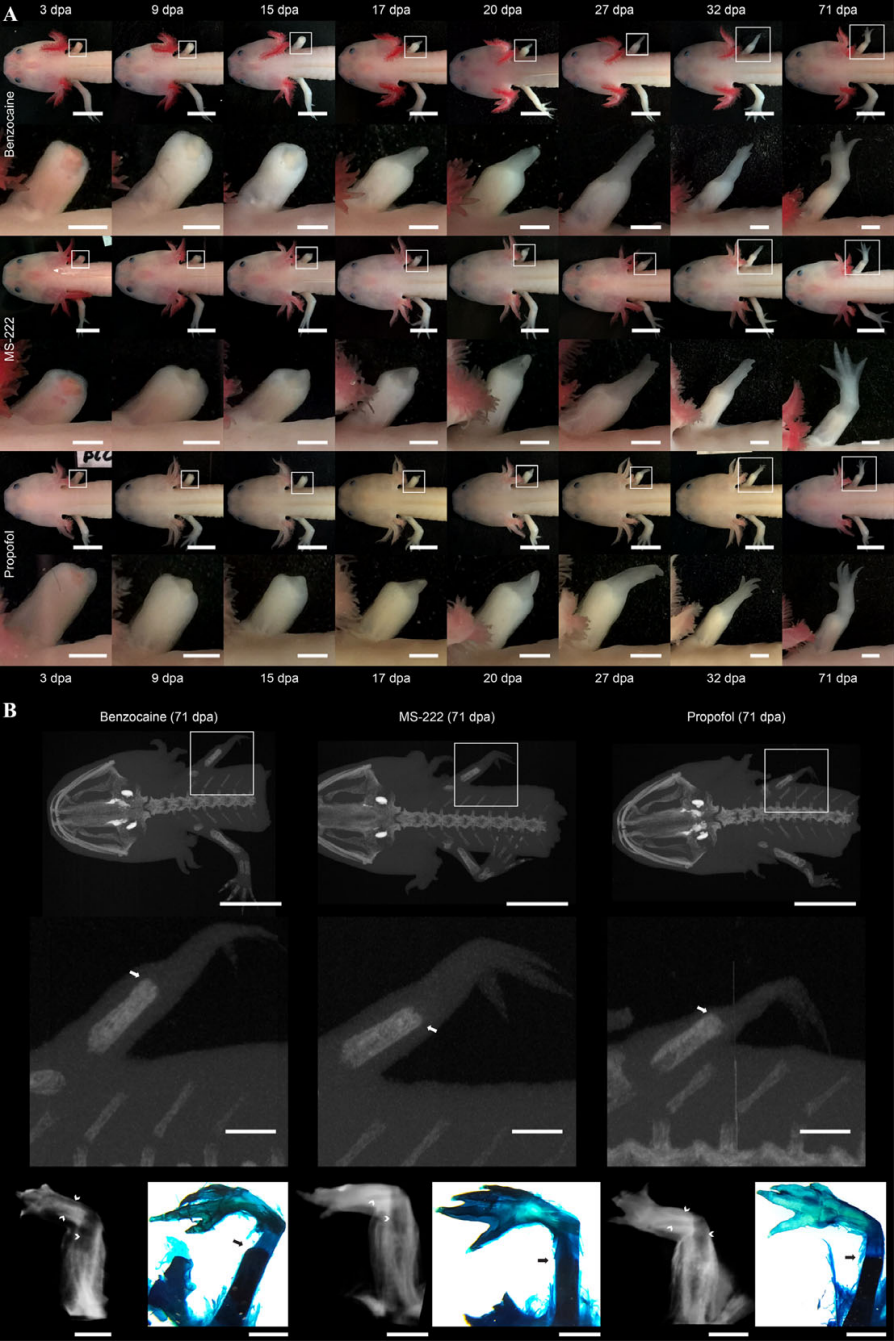
**Effect of benzocaine, MS‐222, and propofol on gross limb regeneration. (**A) Representative photographic time series of regenerating front limbs of axolotls repeatedly anesthetized with either benzocaine (top panel), MS‐222 (middle panel), or propofol (lower panel). Scale bars on main images represent 10 mm and on magnified inserts 2 mm. (B) Representative maximum intensity projection of μCT scans of axolotl repeatedly anesthetized with either benzocaine (left panel), MS‐222 (middle panel), or propofol (right panel) at 71 days post amputation. Lower panel displays μCT images of soft‐tissue iodine stained limb samples and Alcian Blue and Alizarin Red stained whole mounts revealing the formation of calcified and non‐calcified bone structures in the regenerate. Arrows indicate the most distal location of calcified bone in the regenerate; chevrons indicate non‐calcified bone structures in the regenerate. Scale bars on main images represent 10 mm and on magnified inserts 2 mm. dpa, days post amputation

Gross limb regeneration progressed according to the stages originally defined by Tank and colleagues irrespective of the type of anesthetic applied (Fig. 3A) (Tank, Carlson, & Connelly, 1976). Computed microtomography (μCT) inspection and whole‐mount Alcian Blue and Alizarin Red staining of the final regenerate revealed no qualitative differences in both calcified and non‐calcified bone morphology between the different treatments (Fig. 3B). At the time of terminating the animals for this experiment and performing μCT imaging (71 days post amputation), bone mineralization of the newly regenerated limb bones had not commenced in any of the three treatment groups (Fig. 3B).

## 3 DISCUSSION

The axolotl is a powerful animal model for intrinsic tissue regeneration in tetrapods, and considering that most in vivo procedures in the field of regenerative biology require some form of anesthesia it is important to consider the most appropriate anesthetic for a specific investigation. The inspiration for this study came from the potential drawbacks of the traditional axolotl anesthetics, benzocaine and MS‐222, when used in neurological and muscular examinations. Our study demonstrated that propofol was applicable as a general anesthetic in the axolotl; furthermore it could be administered as an immersion anesthetic and when applied at the correct concentration provided a recovery time comparable to benzocaine and MS‐222.

An interesting finding was the relatively small effect of propofol on cardiac function compared to that of benzocaine and MS‐222 (Fig. 1). Except for a small increase in SV in recovering axolotls, propofol did not affect HR whereas an increased HR was found in animals subjected to benzocaine or MS‐222. This finding may have important applications in cardiophysiological investigations or heart regenerative studies in which heart function must be monitored over time. We observed a pronounced vasodilative effect of both benzocaine and MS‐222 compared to that of propofol (can be recognized by comparing the skin/gill color of benzocaine and MS‐222 anesthetized animals [pink/red] to that of propofol anesthetized animals [pale] in Fig. 3A, reflecting that of unanesthetized animals). This vasodilation could in part, and as an addition to the potential beta‐1 adrenergic agonistic effect of benzocaine and MS‐222, explain the need for increased cardiac output in benzocaine or MS‐222 anesthetized animals to maintain blood pressure; however, we did not quantitatively test this hypothesis. Additionally, we found that the intracardiac blood oxygen saturation level was similar in benzocaine and propofol anesthetized animals, ruling out potential ischemic effects of propofol compared to benzocaine. Oxygen uptake at hyperoxia, however, was slightly higher in the benzocaine anesthetized animals which might be related to the general vasodilation in gills and skin augmenting uptake of ambient oxygen.

Propofol proved highly applicable for EMP investigations under deep anesthesia, circumventing the minor inhibition of action potential propagation and less deep anesthesia of MS‐222 and especially the pronounced inhibition of benzocaine. Although EMP amplitude was not significantly different between animals anesthetized with propofol and MS‐222 it is worth noting that the involuntary movements associated with motor cortex stimulation in the less deep MS‐222 anesthesia are likely to be inapplicable in experiments more sensitive to movements, e.g. direct electrical measurements on the surgically exposed spinal cord. On the other hand, propofol allowed for both deep anesthesia even during heavy stimulation of the motor cortex and nerve signal propagation. Propofol has previously been suggested to have dose‐dependent EMP depressing properties in humans (Kalkman et al., 1992), which may occur in axolotls and other amphibians. However, at the concentration applied here EMP signals were easily detectable.

Axolotls are often used in limb regenerative experiments; therefore suggesting the use of propofol as an anesthetic without evaluating any potential effect on the progress of limb regeneration, at least on a gross morphological level, would seem negligent. We found that repeated use of propofol did not result in any qualitative differences in the limb regenerative process that replicates the same steps of blastema, early/medium/late bud, palate and digital outgrowth as has been described previously (Tank et al., 1976), and with the same timing as benzocaine and MS‐222 (Fig. 3).

Propofol is a sedative/hypnotic agent and is not considered analgesic. Overall very little is known about analgesics in amphibians (Stevens, 2011). If using propofol to induce anesthesia during more invasive procedures, additional analgesics may be considered.

In our experience the time to anesthesia and the duration of anesthesia are dependent on the size of the axolotl when applying both benzocaine and MS‐222. This is likely to be true for propofol as well, and although we settled for a uniform size of animals and a single dose of propofol in this study for consistency throughout our experiments, for applying to animals of different sizes than described here and in experiments in which longer periods of anesthesia are required it is essential to perform a preliminary dose−response analysis to optimize the dose required for the particular experiment and size of animals.

It is worth mentioning that, when applying a potent anesthetic such as propofol, additional precautions may be needed to that of more harmless local anesthetics such as benzocaine and MS‐222. Cutaneous uptake of propofol has been described in rats, and pulmonary uptake of aerosolized propofol has been described in humane health personnel (McAuliffe et al., 2006; Merlo, Goldberger, Kolodner, Fitzgerald, & Gold, 2008; Takahashi et al., 2005).

This study focused solely on the use of propofol as an alternative anesthetic in the axolotl, and we demonstrate that this anesthetic may be used with advantages in some defined types of experiments. Likewise, it may be worth considering and testing propofol as an anesthetic in experiments with other amphibians and fish (e.g., the zebrafish) in which the action potential decreasing and HR stimulating effects of benzocaine and MS‐222 may be undesirable.

## 4 METHODS AND MATERIALS

### 4.1 Ethics statement

The procedures carried out in this study were in accordance with the national Danish legislation for care and use of laboratory animals and the experiments were approved by the Danish National Animal Experiments Inspectorate (protocol# 2015‐15‐0201‐00615).

### 4.2 Animals

Animals used in this study were Mexican axolotls (*Ambystoma mexicanum*) (mean body mass ± STD 10.87 g ± 2.35 g; snout to tail length ± STD 11.80 cm ± 0.92 cm) obtained from a commercial breeder (Exoterra GmbH, Holzheim, Germany). Animals were housed individually in plastic containers with a 10 cm water depth and a 930 cm^2^ surface area with regular water change and a 12 h:12 h light:dark cycle. They were fed every second day with protein enriched trout pellets.

### 4.3 Anesthesia

All experiments were repeated using propofol, benzocaine, and MS‐222, except during the photoacoustic measurements of intracardiac oxygen saturation, in which only benzocaine and propofol were tested. In the literature, various concentrations of benzocaine and MS‐222 have been applied to obtain anesthesia in the axolotl spanning an order of magnitude (e.g., McCusker & Gardiner, 2013, 0.1% MS‐222; Quiroz et al., 2014, 0.1% benzocaine; Khattak et al., 2014, 0.03% benzocaine; McHedlishvili et al., 2012, 0.01% benzocaine). We settled on a concentration of benzocaine (Sigma‐Aldrich, CAS# 94‐09‐7) of 200 mg/L (0.02%), which was within the range previously reported and facilitated full anesthesia after 30–60 min (depending on the level of agitation) of immersing the animal in anesthetic containing solution and a complete recovery within 60 min after subsequent immersion in normal aquarium water. A similar concentration of MS‐222 (Sigma‐Aldrich, CAS# 886‐86‐2) was selected as it is structurally very similar to benzocaine. To determine the concentration of propofol (Propofol B, Braun, Melsungen, Germany) needed to acquire a comparable degree of anesthesia and recovery time as for benzocaine and MS‐222, five animals were anesthetized in increasing concentrations of propofol (range 0.3−100 mg/L) with at least 1 day of recovery between each exposure. A comparable level of anesthesia was reached at 3.3 mg/L propofol. We observed that, whereas the time to the induction of general anesthesia varied using benzocaine or MS‐222, depending on the level of agitation and especially gill movement of the animal, propofol showed a much more consistent effect. Therefore, the animals were exposed to 30 min of propofol or 1 h of benzocaine/MS‐222 in subsequent experiments to obtain comparable levels of anesthesia and recovery times. These regimes of anesthesia were employed to test the effect of benzocaine, MS‐222, and propofol on the three separate measures, cardiovascular function, neurophysiological function, and gross limb regeneration.

### 4.4 Cardiovascular experiments

To assess the effect of benzocaine, MS‐222, and propofol on cardiovascular function, six axolotls (body mass ± STD 10.09 g ± 2.35 g; snout to tail length ± STD 10.93 cm ± 0.98 cm) were anesthetized with each agent with 1 week of restitution in between. Cardiac function was assessed by transthoracic echocardiography (TTE) using a Vevo 2100 (VisualSonics, Toronto, Canada) ultrasound system connected to a 50 MHz MS700 transducer. Each animal was placed in 1 L of water in a custom designed Styrofoam container with a thin plastic film bottom, enabling TTE of the unanesthetized and undisturbed animal in the natural prone position (Fig. 1A). The animal was unable to see and hear the operator during the experiment, and the transducer was moving in a gel drop hanging underneath the plastic film, thereby minimizing any movements caused by the translation of transducer. The animal was given 30 min to rest before recordings. A baseline measurement was made prior to adding the respective anesthetic to the water. The diluted anesthetic was applied using a long catheter to ensure that the animal was not disturbed by the nearby presence of a human being. TTE measurements were made every tenth minute until the animal showed signs of general anesthesia, i.e., lack of movement and gill movement. Anesthesia was managed for a minimum of 30 min for propofol and a minimum of 60 min for benzocaine and MS‐222 after which full anesthesia was ensured by checking flight reflexes. Following the final TTE measurement at full anesthesia, water was changed twice to fresh water by siphoning in order to wash out the remaining anesthetic in the container, and TTE measurements were continued every tenth minute for at least 60 min or until signs of full recovery were observed, i.e. coordinated movements. The HR was obtained from TTE pulsed wave Doppler‐mode measurements (Fig. 1B) and related to baseline values. SV was found from two‐dimensional brightness mode TTE by assuming that the ventricle of the axolotl heart can be described as a sphere (Fig. 1C) and using the geometrical equation

SV =43π CSA ( diastole )π3−43π CSA ( systole )π3

where CSA refers to the cross‐sectional area of the ventricle, either in diastole or systole.

To investigate the effect of specific anesthetics on intracardiac blood oxygen saturation, three axolotls (body mass 13.83 ± 0.32 g; length 12.53 ± 0.57 cm) underwent transthoracic photoacoustic imaging using a Vevo 2100 LAZR system (VisualSonics). The signal amplitude and the effective attenuation coefficient were extracted from the photoacoustic signals as a function of wavelength to provide photoacoustic spectra of the blood, and from these the relative concentrations of oxy‐ and deoxy‐hemoglobin (blood oxygen saturation) were estimated using built‐in software on the Vevo 2100 LAZR system.

Photoacoustic imaging was applied on animals anaesthetized with benzocaine and propofol with a full day of recovery between measurements. Axolotls were anesthetized with the respective anesthetic, and blood oxygen saturation in the ventricle was obtained after which oxygen uptake in the anesthetized state was tested by increasing the ambient oxygen saturation to 100% (bubbling the water with pure O_2_). Photoacoustic measurements were performed every 3 s until the intracardiac oxygen saturation reached a stable level after 15 min.

### 4.5 Neurophysiological experiments

To investigate if propofol was applicable as an anesthetic for measuring EMPs, nine axolotls (body mass ± STD 11.00 g ± 2.19 g; snout to tail length ± STD 11.57 cm ± 0.97 cm) were anesthetized with benzocaine, MS222, and propofol, respectively, with 1 day of restitution in between. An EMP examination was performed using transcranial stimulation via penetrating stainless steel electrodes placed transcutaneously 7 mm caudal to the eyes, just superficially to the motor cortex. Stimulation was supplied with a WIP A310 Accupulser signal generator through a WIP A365 stimulus isolator, controlled by a laptop and a National Instruments USB 6152 and custom Labview software (Fig. 2A). A stimulus train of 8 × 10 V pulses of 10 μs pulse duration and 100 μs inter‐pulse interval was applied through the stimulation electrodes to achieve sufficient amplitude of the EMP through spatial summation of the stimulus pulses. Each stimulus train was followed by a 250 ms recording window. In order to improve the signal‐to‐noise ratio each recording was constructed from the average of 30 individual stimulations. Stainless steel recording electrodes were placed intramuscularly and caudally to the hind limbs, facilitating EMP recordings of the lateral tail muscles. The EMPs were amplified 100‐fold using a Grass P50 preamplifier before being digitized by an NI USB 6152 DAQ. The stimulation experiments were performed in a grounded Faraday cage to further improve the signal‐to‐noise ratio.

In order to compare EMPs recorded using different anesthetics, the stimulus responses were measured from the voltage peak‐to‐peak amplitudes, recorded at 25 ms and 100 ms after the onset of stimulation.

### 4.6 Limb regeneration experiments

To investigate potential gross effects of repeated use of propofol as an anesthetic compared to benzocaine and MS‐222 in a limb regeneration setup, three groups of three axolotls (body mass ± STD 11.90 g ± 1.71 g; snout to tail length ± STD 12.19 cm ± 0.72 cm) were exposed to a right front limb amputation with subsequent bone trimming. Each group was anesthetized using benzocaine, MS‐222, or propofol during surgery and subsequently at 3, 9, 15, 17, 20, 27, 32, and 71 days post amputation. At these time points, the level of gross limb regeneration was documented by photography. At 71 days post amputation when a miniature limb had been formed in all animals, μCT imaging of unstained and iodine stained (as described by Metscher, 2009) limb samples was applied to evaluate potential differences in bone regeneration using a Scanco Medical XtremeCT system (Scanco, Brüttisellen, Switzerland), where image acquisition was performed with the following parameters: 0.041 × 0.041 × 0.041 mm^3^ voxel size; 59.4 kVp tube voltage; 119 μA s tube charge, and an acquisition time of 65 min. Subsequently, whole‐mount limb samples were stained with Alcian Blue and Alizarin Red (as described by Horton & Maden, 1995) to validate cartilage formation and bone calcification patterns observed using μCT imaging.

### 4.7 Statistics

For the neurophysiological data Mann−Whitney *U* tests were conducted on peak‐to‐peak amplitude. For all other experiments comparison between groups was performed using Student's *t* test for paired data with *p* < 0.05 as the significance level. Error bars in all figures represent 95% confidence intervals.
